# Effect of maternal sleep, physical activity and screen time during pregnancy on the risk of childhood respiratory allergies: a sex-specific study

**DOI:** 10.1186/s12931-020-01497-8

**Published:** 2020-09-03

**Authors:** Yiting Chen, Jiajun Lyu, Yuanqing Xia, Jianzhen Zhu, Shilu Tong, Yong Ying, Jiajie Qu, Shenghui Li

**Affiliations:** 1 School of Public Health, Shanghai Jiao Tong University School of Medicine, 227 South Chongqing Road, Huangpu District, Shanghai, 200025 China; 2 School of International and Public Affairs, Shanghai Jiao Tong University, Shanghai, China; 3 Shanghai Children’s Medical Center, Shanghai Jiao Tong University School of Medicine, Shanghai, China; 4 School of Public Health and Social Work, Queensland University of Technology, Brisbane, Australia; 5 Shanghai Municipal Education Commission, 100 Dagu Road, Huangpu District, Shanghai, 200003 China; 6 MOE-Shanghai Key Laboratory of Children’s Environmental Health, Shanghai Jiao Tong University School of Medicine, Shanghai, China

**Keywords:** Allergic disease, Maternal behavioral characteristics, Pregnancy, Preschool children

## Abstract

**Background:**

Early life exposure in the uterus had a long-term effect on children’s health. As the prevalence of allergies is increasing with a remarkable sex difference, very few studies have traced back to their early origins. We sought to investigate if maternal behavioral exposure, herein sleep, physical activity, and screen time during pregnancy is associated with childhood respiratory allergies. The sex difference would be examined.

**Methods:**

Six thousand two hundred thirty-six mother-child pairs from Shanghai Children Allergy Study (SCAS) were enrolled, The International Study of Asthma and Allergies in Childhood questionnaire was adopted to evaluate respiratory allergic diseases.

**Results:**

14.6, 16.2, and 21.0% of children had asthma, wheeze, and allergic rhinitis, respectively. Maternal short sleep duration, lack of physical activity, and too much screen exposure during pregnancy could increase the risk of childhood respiratory allergies, however, the significance was found only in males. Moreover, a dose-response trend was clearly shown, any two of the three combined could increase the risk (OR,1.921; 95% CI,1.217–3.033), and the coexistence of all three further amplified the risk (OR,2.412; 95% CI,1.489–3.906). The findings can be verified in allergen test subgroup and each single type of respiratory allergies in most cases.

**Conclusions:**

Maternal unhealthy behaviors during pregnancy could increase the risk of childhood respiratory allergies with a dose-response pattern. Males were more susceptible to the association. The identification of modifiable maternal risk behaviors lies in the emphasis of intervention in early life to face up increasing childhood allergies.

## Introduction

The prevalence of childhood allergic diseases continues to rise with the development of economy and urbanization all around the world. China has the largest population in the world, and quantities of rural people flooded into cities with the acceleration of urbanization since 1980s [1, 2]. Shanghai is one of the fastest urbanizing cities in China, where the prevalence of childhood asthma increased almost fivefold from 1990 to 2011 [3]. Data from a national survey covering 10 cities across China demonstrated that childhood allergic diseases, except for wheezing, were most prevalent in Shanghai [4]. Although numerous potential environmental risk factors have been proposed, it is still far away to explain the rapid rise. It has been a general recognition that early-life in utero is a special period for imprinting, programming and the establishment of epigenome [5]. Accumulating studies were providing evidence that early life exposure has not only short term effects on fetal growth but also long-term impacts on individual’s health and disease susceptibility in later life [5].

To date, there are several shreds of evidence suggesting that early life exposure during pregnancy may be implicated in the development of the immune system. Rodent model has long shown that air pollution exposure during pregnancy increased the susceptibility of neonates to allergic asthma [6]. A meta-analysis on epidemiology evidence similarly suggested that prenatal exposures to NO_2_, SO_2_, and PM_10_ increased the risk of wheezing and asthma in childhood [7]. Besides, the association between prenatal nutritional exposures and childhood allergies were also proposed in the last two decades [8]. Meanwhile, a few studies reported that maternal behavior choice was involved in childhood allergies [8, 9]. Early in 1998, Boston conducted a prospective birth cohort study in 505 mother-infant pairs, revealing that maternal smoking during pregnancy could increase the risk of childhood wheezing in the first year of life [9]. A newly published cross-sectional study also reported that maternal smoking was associated with a higher risk of asthma in their offspring [10]. However, data on maternal lifestyle such as sleeping and physical activity are largely lacking. With rapid modernization, sleep duration, physical activity, and screen using are indeed lifestyle choices people face every day. It has been a growing concern that unhealthy and sedentary lifestyle, including chronic sleep loss, inactivity, and excessive screen exposure, is quite common [11]. A national school-based health survey in Greece revealed that physical activity during pregnancy was significantly associated with childhood obesity [12]. A prospective study found that sleep difficulties during pregnancy affected the social development of their offspring when they were 1 year old [13]. However, none of the studies have investigated whether maternal lifestyle factors during pregnancy had an impact on childhood allergies.

Sex dimorphism has long been known to childhood allergies [3, 14], but the sex difference by the same risk exposure has rarely been studied. The genetic background has considered being a risk factor for allergies [15]. Therefore, attention to sex differences, taking familial heredity into account, would help us deeply understand the childhood allergy, which should be the basis of personalized allergy intervention.

A citywide study was conducted in Shanghai to explore the association of maternal lifestyle factors, including sleep duration, daily physical activity, and screen exposure, with the risk of respiratory allergies in their offspring. Specifically, sex differences will be a focus.

## Methods

### Study participants and ethics statement

Shanghai Children Allergy Study (SCAS) is an ongoing study, aiming to explore the epidemic characteristics of childhood allergies and to formulate the intervention strategy for children allergy in Shanghai. The study was sponsored by the Shanghai Education Commission. We collaborate with Shanghai Children’s Medical Center to carry out the project research. This cross-sectional baseline study was conducted during April–June 2019, using a multi-stage and multi-strata sampling approach. Among the total 9 urban areas and 8 suburban/rural areas in Shanghai, 4 urban areas (Xuhui, Putuo, Yangpu, and Pudong New Area) and 4 suburban/rural areas (Minhang, Jinshan, Qingpu, and Chongming) were randomly sampled. In all sampled areas, 31 kindergartens were randomly selected. The investigation purpose was explained ahead to the principals and teachers. To obtain parental permission, the purpose was conveyed to parents through parents’ meetings, in which voluntary and anonymous participation was stressed. The informed consent form was then obtained from parents. Each child’s mother completed the questionnaire on behalf of herself and her child.

Overall, 6389 children were recruited to participate in this baseline survey. Among them, 6237 (97.62%) completed the questionnaires. After the exclusion of 1 missing data on allergic diseases, the valid sample of this study consisted of 6236 mother-children pairs (defined as All Population Group).

The ethical application and the consent procedure of this study were approved by the Ethics Committee of Shanghai Jiao Tong University School of Medicine.

### Maternal behavioral variables

A brief Maternal Lifestyle Questionnaire (MLQ) was applied to evaluate maternal lifestyles during pregnancy based on the literature reviewed [16–18]. Daily behavioral choice during pregnancy included the duration of sleep per day, the frequency of physical activity (never/occasionally, 1-3 h./week, 3-6 h./week, ≥1 h./day), and the frequency of phone/computer/television exposure (< 2 h./day, 2-4 h./day, 4-8 h./day, ≥8 h./day) on average. The optimal duration of sleep for the pregnant woman is not yet known, the cutoff for short sleep duration was defined as less than 8 h/day in this study with the reference of most studies [18, 19]. Meanwhile, 8 h/day was close to the 25th percentile of the distribution of total sleep duration during pregnancy in our dataset. The frequency of daily physical activity was classified into two groups: < 1 h./day vs. ≥1 h./day; and screen exposure was also classified into two groups: ≥2 h./day vs. < 2 h./day.

### Ascertainment of allergic diseases

The International Study of Asthma and Allergies in Childhood (ISAAC) questionnaire was applied to detect respiratory allergies [20, 21]. The Cronbach’s alpha coefficient of the ISAAC allergic questionnaire in our sampled children was 0.91. The intra-class correlation coefficient of retest reliability at intervals of 2 weeks was 0.94. Validity presented by Kaiser-Meyer-Olkin (KMO) was 0.94 and the high validity among preschool children has been confirmed [22]. All questions were answered by yes or no. For the assessment of asthma, the question was asked: “Has your child ever been diagnosed with asthma?”. We further assessed the current wheezing by questions: “Has your child had any wheezing, or breathing difficulties in the last 12 months?” or “Has your child ever experienced wheezing or whistling in the chest in the last 12 months?”. Two questions were utilized for allergic rhinitis diagnosis: “Has your child ever been diagnosed with allergic rhinitis?” and “Has your child ever sneezing, runny nose, stuffy, itchy or itchy eyes in the last 12 months not due to having a cold or flu?”. Children were screened out one of above three types of respiratory allergies were considered to be Screened Positive (SP).

In addition to using the ISAAC questionnaire to screen respiratory allergies, we further collected information on allergen tests. Of those who took part in the allergen test through skin prick test (SPT), immunoglobulin E (IgE) and the others, we further asked the children about more detailed information regarding results from allergen test: “Did the allergen test is positive, if so, which of the following allergens?” Common environmental allergens were listed, including dust mite, mold, pollen, mugwort, ragweed, cat/dog hair. Among children with screened respiratory allergies positive, those who tested positive for any of inhaled allergens were further regarded as Screened and Test Positive (STP), and those were screened negative and never went through any allergen test were set as reference group. Eventually, a total of 3877 participants were in the Allergen Test Subgroup.

### Confounding variables

#### Demographic characteristics

Demographic characteristics included age and childhood overweight/obesity (yes/no), child’s sleep duration on weekdays (< 10 h./day, ≥10 h./day), child’s sleep duration on weekends (< 10 h./day, ≥10 h./day), child’s exercise frequency (< 1 h./day, ≥1 h./day), child’s screen exposure frequency (< 5 times./week, ≥5 times./week). BMI (body mass index) for age and sex-specific percentile over 85% was implemented to define the overweight/obesity [23]. Family background contained household incomes (< 4000, 4000–8000, > 8000), family structures (single parent family, nuclear family, extended family), both mother and father’s educational levels (primary education, secondary education, college and above), and first-degree relative with allergies (yes/no).

#### Obstetric characteristics and health status of parents

Obstetric characteristics included gestational weeks (premature delivery, post-delivery, and term delivery), delivery modes (cesarean delivery, vaginal delivery/midwifery), and full breastfeeding over 6 months (yes/no). Health status of parents contained maternal smoking exposure status (yes/no), maternal drinking habits (yes/no), gestational hypertension (yes/no), gestational diabetes (yes/no), gestational anemia (yes/no), maternal pre-pregnancy overweight/obesity (yes/no), mother’s age at delivery (≥30, 25–29, ≤24). Those pre-pregnancy maternal body mass index above 25 (including 25) was defined as maternal pre-pregnancy overweight/obesity [24].

#### Environmental exposure

Four questions on the environmental conditions, asking whether there were farms or other sources of pollution within a five-minute walk from home, including farmlands and orchards (yes/no), chemical emission sources (yes/no), smog-emitting factories (yes/no), large garbage dumps (yes/no).

### Statistical analysis

Statistical descriptions were made utilizing the percentage for categorical variables. The univariate logistic regression was applied to compare differences between groups.

Univariate logistic regression was implemented to calculate the unadjusted odds ratios (OR) and 95% confidence intervals (CI), thus evaluating the relationship between maternal behavioral characteristics and allergic diseases. In All Population Group, ‘1’ for children with screened allergies positive and ‘0’ for children screened allergies negative. In Allergen Test Subgroup, children with both positive in allergen tests and screened respiratory allergies were defined as ‘1’, and who had neither been screened with any allergy nor took part in allergen tests were defined as ‘0’. Adjustments of confounding factors were made by the multivariate regression models followed by a two-step procedure: Model I only adjusted for demographic characteristics; in Model II, obstetric characteristics and health status of parents were further adjusted.

All other analyses were performed with the Statistical Package for the Social Sciences (SPSS) (IBM-SPSS Statistics version 23.0, Inc., Chicago, IL). The statistical significance level was set at *p*-value <.05 (two-sided).

## Results

### Descriptive analysis

**Fig. 1:**
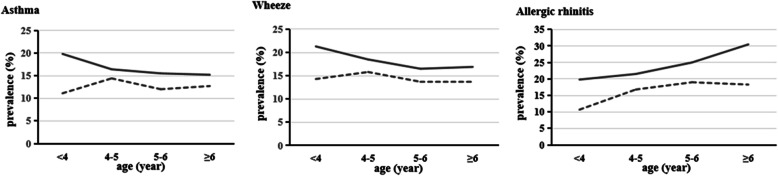
Age and sex-specific prevalence of childhood respiratory allergies Solid lines represent males, dotted lines represent females

The study sample included 6236 children (3289 males vs. 2947 females), aged 5.16 years (SD = 0.88, ranging from 2.02 to 6.99 years old). 14.6, 16.2, and 21.0% of children had asthma, wheeze, and allergic rhinitis, respectively. The demographic characteristics of the 3877 participants who took the allergen test were similar to those of 6236 (Table 1). Among allergic children, there is no sex difference in maternal exposure to unhealthy behaviors (Table 2). Figure 1 plots the prevalence of asthma, wheeze, and allergic rhinitis with increasing age by different sex. It can be seen that**,** compared to females, males are more susceptible to all three respiratory allergies.

**Table 1:** Sample characteristics, stratified by gender

	All Population Group			Allergen Test Subgroup		
	Boy (***n*** = 3289)	Girl (***n*** = 2947)	χ^**2**^/t	Boy (***n*** = 1975)	Girl (***n*** = 1902)	χ^**2**^/t
	***n*** (%)	***n*** (%)		***n*** (%)	***n*** (%)	
**Demographic characteristics**						
Age (years); mean (SD)	5.16 (0.87)	5.15 (0.88)	0.318	5.15 (0.87)	5.14 (0.89)	0.251
Childhood overweight/ obesity	666 (20.3%)	469 (16.0%)	19.768***	418 (21.3%)	298 (15.7%)	19.508***
Child’s sleep duration on weekdays (≥10 h./day)	557 (16.9%)	496 (16.8%)	0.013	377 (17.1%)	309 (16.3%)	.466
Child’s sleep duration on weekends (≥10 h./day)	960 (29.3%)	980 (33.3%)	12.001^***^	612 (31.1%)	620 (32.7%)	1.167
Child’s screen exposure frequency (≥5 times./week)	436 (13.3%)	400 (13.6%)	0.134	250 (12.7%)	250 (13.1%)	0.204
Child’s exercise frequency (≥1 h./day)	1450 (44.1%)	1225 (41.6%)	4.025^*^	909 (46.0%)	803 (42.2%)	5.694^*^
Family income ^**#**^			0.265			0.993
> 8000RMB	1705 (51.8%)	1519 (51.5%)		1030 (52.2%)	963 (50.6%)	
4000- 8000RMB	1228 (37.3%)	1117 (37.9%)		727 (36.8%)	728 (38.3%)	
< 4000RMB	356 (10.8%)	311 (10.6%)		218 (11.0%)	211 (11.1%)	
Family structure			0.587			0.001
Single parent	89 (2.7%)	81 (2.7%)		51 (2.6%)	49 (2.6%)	
Nuclear family	1864 (56.7%)	1697 (57.6%)		1141 (57.8%)	1098 (57.7%)	
Extended family	1336 (40.6%)	1169 (39.7%)		783 (39.6%)	755 (39.7%)	
Mother’s education level			6.026*			4.590
College and above	2442 (74.2%)	2243 (76.1%)		1464 (74.1%)	1458 (76.7%)	
Secondary education	509 (15.5%)	454 (15.4%)		310 (15.7%)	285 (15.0%)	
Primary education	338 (10.3%)	250 (8.5%)		201 (10.2%)	159 (8.4%)	
Father’s education level			2.524			2.290
College and above	2439 (74.2%)	2234 (75.8%)		1456 (73.7%)	1440 (75.7%)	
Secondary education	564 (17.1%)	482 (16.4%)		347 (17.6%)	316 (16.6%)	
Primary education	286 (8.7%)	231 (7.8%)		172 (8.7%)	146 (7.7%)	
First-degree relative with allergies	897 (27.3%)	767 (26.0%)	1.234	471 (23.8%)	427 (22.5%)	1.064
**Obstetric and parental health condition**						
Gestational weeks			2.022			2.113
Premature delivery	248 (7.6%)	202 (6.9%)		142 (7.2%)	124 (6.6%)	
Post-term delivery	128 (3.9%)	130 (4.5%)		69 (3.5%)	81 (4.3%)	
Term delivery	2890 (88.5%)	2589 (88.6%)		1754 (89.3%)	1679 (89.1%)	
Delivery mode			6.525*			7.401**
Cesarean delivery	1717 (52.2%)	1443 (49.0%)		1025 (51.9%)	904 (47.5%)	
Vaginal delivery/ midwifery	1572 (47.8%)	1504 (51.0%)		950 (48.1%)	998 (52.5%)	
Mother’s age at delivery			0.612			1.101
≥ 30	1286 (39.6%)	1139 (39.2%)		765 (39.2%)	731 (39.0%)	
25–29	1458 (44.9%)	1332 (45.9%)		875 (44.8%)	864 (46.1%)	
≤ 24	500 (15.4%)	433 (14.9%)		311 (15.9%)	278 (14.8%)	
Full breastfeeding ≥6 month	1885 (57.3%)	1754 (59.5%)	3.112	1151 (58.3%)	1117 (58.7%)	0.081
Gestational smoking exposure	71 (2.2%)	63 (2.1%)	0.003	35 (1.8%)	39 (2.1%)	0.401
Gestational drinking	84 (2.6%)	2878 (97.7%)	0.294	47 (2.4%)	46 (2.4%)	0.006
Gestational hypertension	60 (1.8%)	85 (2.9%)	7.690**	33 (1.7%)	45 (2.4%)	2.374
Gestational diabetes	149 (4.5%)	142 (4.8%)	0.290	87 (4.4%)	82 (4.3%)	0.020
Gestational anemia	189 (5.7%)	200 (6.8%)	2.875	113 (5.7%)	124 (6.5%)	1.075
Pre-pregnancy overweight/obesity	328 (10.0%)	267 (9.1%)	1.453	203 (10.3%)	163 (8.6%)	3.269
**Environmental condition**						
Farmlands and orchards	574 (17.5%)	518 (17.6%)	.017	329 (16.7%)	305 (16.0%)	0.274
Chemical emission sources	82 (2.5%)	87 (3.0%)	1.242	45 (2.3%)	48 (2.5%)	0.249
Smog-emitting factory	79 (2.4%)	91 (3.1%)	2.758	41 (2.1%)	56 (2.9%)	2.995
Large garbage dump	232 (7.1%)	208 (7.1%)	.000	136 (6.9%)	129 (6.8%)	.898
**Respiratory allergic disease**						
Asthma	533 (16.2%) ^a^	380 (12.9%) ^a^	13.635***	150 (7.6%) ^b^	78 (4.1%) ^b^	21.370***
Wheeze	586 (17.8%) ^a^	427 (14.5%) ^a^	12.650***	165 (8.4%) ^b^	90 (4.7%) ^b^	20.693***
Allergic rhinitis	802 (24.4%) ^a^	509 (17.3%) ^a^	47.357***	225 (11.4%) ^b^	129 (6.8%) ^b^	24.818***

* *p* < 0.05, **p < 0.01, ***p < 0.001; ^#^Family income is calculated as RMB/person/month, RMB is China’s currency (yuan)

^as^ Children only with screened respiratory allergies. ^b^ Children with both screened respiratory allergies and allergen test positive

**Table 2:** Maternal behavioral characteristics among children with respiratory allergies, stratified by gender

	SP (***n*** = 1904)		χ^**2**^/t	STP (***n*** = 464)		χ^**2**^/t
	boy	girl		boy	girl	
**Individual factor**						
**Sleep duration**			2.093			.374
< 8 h./day	340 (30.4%)	213 (27.3%)		85 (29.0%)	44 (26.3%)	
≥ 8 h./day	778 (69.6%)	566 (72.7%)		208 (71.0%)	123 (73.7%)	
**Physical activity**			.095			2.110
< 1 h./day	936 (83.6%)	651 (83.0%)		253 (86.3%)	139 (81.3%)	
≥ 1 h./day	184 (16.4%)	133 (17.0%)		40 (13.7%)	32 (18.7%)	
**Screen exposure**			1.376			.220
≥ 2 h./day	887 (79.2%)	638 (81.4%)		240 (81.9%)	143 (83.6%)	
< 2 h./day	233 (20.8%)	146 (18.6%)		53 (18.1%)	28 (16.4%)	
**Combined factor index**						
0	30 (2.7%)	22 (2.8%)	.857	4 (1.4%)	2 (1.2%)	1.353
1	249 (22.3%)	170 (21.8%)		60 (20.5%)	42 (25.1%)	
2	607 (54.3%)	437 (56.1%)		169 (57.7%)	91 (54.5%)	
3	232 (20.8%)	150 (19.3%)		60 (20.5%)	32 (19.2%)	

*SP* screened respiratory allergies positive, *STP* both screened respiratory allergies positive and allergen test positive

Maternal behavioral factors included sleep less than 8 h/day, exercise less than 1 h/day, screen time more than 2 h/day

“0” demonstrated that none of three risk factors were occurred; “1” demonstrated that one of these three risk factors was occurred; “2” demonstrated that two of these three risk factors were occurred.; “3” demonstrated that these three risk factors were all occurred

### Maternal behavioral exposure during pregnancy and childhood allergic diseases

#### Individual associations

In Table 3, we performed a sex-stratified analysis and found that maternal short sleep duration, lack of physical activity, and too much screen exposure during pregnancy were associated with an increased risk of childhood respiratory allergies. When the analyses were repeated in Allergen Test Subgroup, very similar results were obtained. However, a significant sex difference was identified, all the above associations were mainly observed in males. Through a two-step adjustment for possible confounding factors, the results were generally kept in most cases. In the final adjusted model (Model II), maternal short sleep duration (OR,1.317; 95% CI:1.102–1.573) and lack of physical activity (OR,1.311; 95% CI:1.066–1.612) were found to be an independent predictor for childhood allergies, however, only in the All Population Group; Among females, by contrast to males, only sleep duration (OR,1.238; 95% CI:1.025–1.495) and screen exposure (OR, 1.421; 95% CI: 1.151–1.754) were shown to be associated with childhood respiratory allergies in the crude model. However, after accounting for confounding factors, the associations were not shown anymore.

**Table 3:** Association of maternal behavioral factors during pregnancy with childhood respiratory allergies

	All Population Group			Allergen Test Subgroup		
	OR (95% CI)			OR (95% CI)		
	Crude Model	Adjusted Model I	Adjusted Model II	Crude Model	Adjusted Model I	Adjusted Model II
**Male**						
**Sleep duration**						
< 8 vs. ≥8 h./day	**1.477 (1.253–1.741)** ^***^	**1.332 (1.117–1.589)** ^***^	**1.317 (1.102–1.573)** ^**^	**1.435 (1.085–1.898)** ^*^	1.208 (0.890–1.640)	1.164 (0.811–1.672)
**Physical activity**						
< 1 vs. ≥1 h./day	**1.525 (1.260–1.846)** ^**^	**1.325 (1.079–1.618)** ^**^	**1.311 (1.066–1.612)** ^*^	**1.887 (1.326–2.686)** ^***^	**1.633 (1.118–2.386)** ^*^	**1.507 (1.022–2.222)** ^*^
**Screen exposure**						
≥ 2 vs. < 2 h./day	**1.467 (1.232–1.748)** ^***^	1.157 (0.954–1.403)	1.189 (0.979–1.445)	**1.645 (1.195–2.264)** ^**^	1.171 (0.823–1.667)	0.966 (0.719–1.298)
**Female**						
**Sleep duration**						
< 8 vs. ≥8 h./day	**1.238 (1.025–1.495)** ^*^	1.098 (0.900–1.339)	1.072 (0.877–1.310)	1.244 (0.863–1.794)	1.044 (0.709–1.540)	1.019 (0.685–1.515)
**Physical activity**						
< 1 vs. ≥1 h./day	1.148 (0.924–1.428)	1.020 (0.811–1.282)	0.994 (0.789–1.253)	0.973 (0.645–1.467)	0.764 (0.491–1.191)	0.757 (0.482–1.190)
**Screen exposure**						
≥ 2 vs. < 2 h./day	**1.421 (1.151–1.754)** ^**^	1.147 (0.914–1.438)	1.181 (0.939–1.485)	**1.602 (1.030–2.492)** ^*^	1.251 (0.781–2.004)	1.256 (0.778–2.027)

*OR* odds ratios, *CI* confidence interval

* *p* < 0.05, **p < 0.01, ***p < 0.001

Model I adjusted for child’s age, childhood overweight/obesity, child’s sleep duration on weekdays, child’s sleep duration on weekends, child’s exercise frequency, children’s screen exposure frequency, family income, family structure, mother’s education, father’s education, family history of allergic diseases

Model II further adjusted for gestational weeks, delivery mode, full breastfeeding, maternal smoking exposure, maternal drinking, gestational hypertension, gestational diabetes, gestational anemia, pre-pregnancy overweight/obesity, mother’s age at delivery, farmlands and orchards near house, chemical discharging near house, factories emitting smoke near house, large garbage dump near house

The analysis was also repeated in each single type of respiratory allergic disease, and similar results were obtained (**Table S****1**). After further considering family history in the first-degree relative, the results were generally repeatable in most cases, however, it seemed that the associations were stronger in those children without an allergic family history (**Table S****2**).

#### Combined associations

**Fig. 2:**
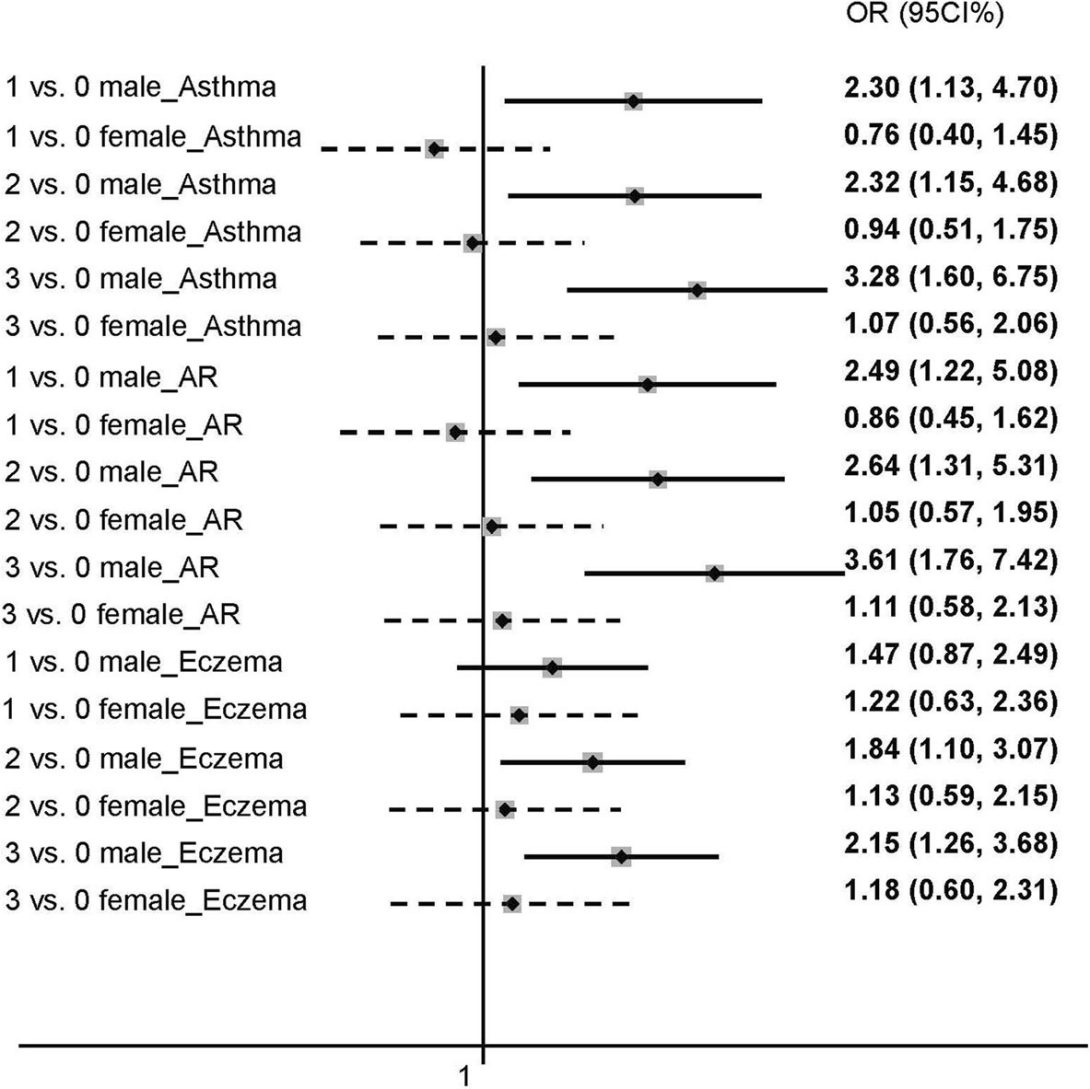
Combined association of maternal behavioral factors and childhood respiratory allergies. AR, allergic rhinitis. Solid lines represent males, dotted lines represent female. Maternal behavioral factors including sleep less than 8 h/day, physical activity less than 1 h/day, screen time more than 2 h/day. “0” demonstrated that none of three risk factors were occurred. “1” demonstrated that one of these three risk factors were occurred. “2” demonstrated that two of these three risk factors were occurred. “3” demonstrated that these three risk factors were all occurred. Controlled for child’s age, childhood overweight/obesity, child’s sleep duration on weekdays, child’s sleep duration on weekends, child’s exercise frequency, children’s screen exposure frequency, family income, family structure, mother’s education, father’s education, family history of allergic diseases, gestational weeks, delivery mode, full breastfeeding, maternal smoking exposure, maternal drinking, gestational hypertension, gestational diabetes, gestational anemia, pre-pregnancy overweight/obesity, mother’s age at delivery, farmlands and orchards near house, chemical discharging near house, smog-emitting factories near house, large garbage dump near house

Table 4 demonstrated the combined associations of three maternal behavioral factors with childhood respiratory allergies with sex as a stratified factor. Children’s risk of respiratory allergies increased with an increase in unhealthy maternal lifestyles during pregnancy. Very similar results were found when the analysis was replicated in the Allergen Test Subgroup. Similar to the individual association, the sex difference of the combination association was significant. After a two-step adjustment, the risk remained mostly in males. In the final adjusted model (Model II), one of the three factors occurred can result in a higher risk of childhood respiratory allergies only in All Population Group (OR,1.645; 95% CI:1.029–2.630); when two of these lifestyles were involved, the significant results were found in All Population Group (OR,1.921; 95% CI:1.217–3.033); as referring to the combination of three unhealthy behavioral factors, the risk further amplified in both All Population Group (OR,2.412; 95% CI:1.489–3.906) and Allergen Test Subgroup (OR,2.217; 95% CI:1.018–4.827). When it came to females, the risk of allergies increased only when two or more of the maternal unhealthy lifestyles concentrated together in the crude model. However, after controlling for confounding factors, no association was revealed. Figure 2 demonstrates the combined association of maternal lifestyle with asthma, wheeze, and allergic rhinitis, respectively.

**Table 4:** Combined association of maternal behavioral factors during pregnancy with childhood respiratory allergies

	All Population Group				Allergen Test Subgroup			
	*N* (%)	OR (95% CI)			*N* (%)	OR (95% CI)		
		Crude Model	Adjusted Model I	Adjusted Model II		Crude Model	Adjusted Model I	Adjusted Model II
**Male**								
**0**	30 (2.7%)	Ref	Ref	Ref	4 (1.4%)	Ref	Ref	Ref
**1 vs. 0**	249 (22.3%)	**1.762 (1.132–2.744)** ^*^	**1.668 (1.046–2.662)** ^*^	**1.645 (1.029–2.630)** ^*^	60 (20.5%)	**3.025 (1.074–8.519)** ^*^	**3.127 (1.068–9.157)** ^*^	**3.063 (1.034–9.073)** ^*^
**2 vs. 0**	607 (54.3%)	**2.508 (1.633–3.853)** ^***^	**1.959 (1.244–3.086)** ^**^	**1.921 (1.217–3.033)** ^**^	169 (57.7%)	**4.903 (1.779–13.513)** ^**^	**3.978 (1.389–11.386)** ^*^	**3.648 (1.261–10.559)** ^*^
**3 vs. 0**	232 (20.8%)	**3.523 (2.243–5.536)** ^***^	**2.464 (1.526–3.981)** ^***^	**2.412 (1.489–3.906)** ^***^	60 (20.5%)	**6.382 (2.256–18.048)** ^***^	**4.171 (1.412–12.317)** ^*^	**3.895 (1.304–11.634)** ^*^
**Female**								
**0**	22 (2.8%)	Ref	Ref	Ref	1 (1.2%)	Ref	Ref	Ref
**1 vs. 0**	170 (21.8%)	1.283 (0.784–2.127)	1.110 (0.661–1.863)	1.041 (0.617–1.755)	42 (25.1%)	3.204 (0.756–13.572)	2.555 (0.587–11.124)	2.677 (0.599-11.954)
**2 vs. 0**	437 (56.1%)	**1.652 (1.015–2.689)** ^*^	1.178 (0.711–1.952)	1.101 (0.662–1.833)	91 (54.5%)	3.385 (0.816–14.039)	2.022 (0.470–8.695)	2.013 (0.457–8.866)
**3 vs. 0**	150 (19.3%)	**1.957 (1.173–3.267)** ^*^	1.298 (0.761–2.214)	1.217 (0.710–2.084)	32 (19.2%)	4.251 (0.995–18.158)	2.272 (0.511–10.101)	2.316 (0.507–10.568)

*OR* odds ratios, *CI* confidence interval, *Ref* reference

^*^* *p* < 0.05, **p < 0.01, ***p < 0.001

Maternal risk behavioral factors included sleep duration less than 8 h, physical activity less than 1 h/day, screen exposure more than 2 h/day; “0” demonstrated that none of three risk factors were occurred. “1” demonstrated that one of these three risk factors was occurred; “2” demonstrated that two of these three risk factors were occurred; and “3” demonstrated that these three risk factors were all occurred

Model I adjusted for child’s age, childhood overweight/ obesity, child’s sleep duration on weekdays, child’s sleep duration on weekends, child’s exercise frequency, children’s screen exposure frequency, family income, family structure, mother’s education, father’s education, family history of allergic diseases

Model II further adjusted for gestational weeks, delivery mode, full breastfeeding, maternal smoking exposure, maternal drinking, gestational hypertension, gestational diabetes, gestational anemia, pre-pregnancy overweight/obesity, mother’s age at delivery, farmlands and orchards near house, chemical discharging near house, smog-emitting factories near house, large garbage dump near house

Similar results were observed when the analysis was repeated in a single type of respiratory allergic disease (**Table S****3**).

## Discussion

This is the first study exploring the associations of maternal lifestyle choice during pregnancy with the risk of childhood respiratory allergic diseases. Our findings demonstrated that all of the three unhealthy maternal lifestyles, including short sleep duration, lack of physical activity and too much screen exposure, were independent risk predictors. Also of concern was significant dose-response trend, the more concentrations of maternal unhealthy lifestyles during pregnancy, the greater risk of respiratory allergies in their children. Moreover, significant sex difference was identified that males were more susceptible to the adverse effects. The results, in most cases, can be verified in the Allergen Test Subgroup and the single type of disease, which further enforced the evidence. The present study drew the attention on the association of maternal behavior exposure with immune development and allergic disease susceptibility in their offspring, in which sex difference should be taken into consideration.

The rapid increase in childhood allergic disease may be related to rapid economic growth or modernization [3]. In developed countries, the prevalence of childhood allergic respiratory symptoms appears to have peaked and stabilized [25]. China, as a developing country, is rapidly urbanizing and the prevalence of childhood respiratory allergies is now on the rise, especially in fast-growing cities such as Shanghai [3]. Allergic diseases was usually higher prevalent in urban area than in rural areas within a geographical region [26], though there existing some inconsistent results [27]. Urbanization is accompanied by a modern style of life, electronic screen exposure is more frequent, sedentary time is increasing while sleeping time is decreasing [11], and it has been proved that pregnant women spend at least half their time on sedentary behaviors [28].

Our results provided new evidence to the hypothesis that a mother’s lifestyle choice during pregnancy could affects offspring’s long-term health, herein childhood respiratory allergies. Previous studies exploring the effect of lifestyle during pregnancy on the long-term health of children largely focused on the neurological and behavioral development [29–32]. A rodent model found that maternal lack of sleep in the third trimester can impair autonomic responses in male offspring with a long-term effect [29]. Cohort data among 83,884 mother-child pairs from five countries revealed that maternal cellphone use during pregnancy increased the risk of hyperactivity in their offspring [30]. Another two prospective studies found that adequate physical activity during pregnancy could promote language development in children [31, 32]. This study, for the first time, focused on maternal lifestyle choice and the susceptibility of childhood allergic disease. Besides, most of the previous studies only assessed one aspect of maternal lifestyles, and their combined effects have never been studied. In our study, a clear dose-response relationship was established, the more exposures to maternal unhealthy lifestyles during pregnancy, the higher risk of childhood respiratory allergies.

Early life exposure in utero can affect the fetal airway and lung development, as well as immune function [33]. As suggested by previous studies, maternal smoking, unqualified diet, vitamin D deficiency and exposure to air pollution should be potential risk factors [7, 34–37]. A retrospective follow-up study was conducted among 1201 Los Angeles women between 3 and 6 months after delivery, where it was shown that children whose mothers ate more fast food during pregnancy had an increased risk of asthma [34]. Although there existed different conclusions [35, 36], a more recent meta-analysis revealed that maternal prenatal vitamin D supplementation was associated with a lower risk of allergic diseases in children [37]. Compared to these studies, we focused on daily lifestyle choices, including sleep duration, physical activity, and screen using, and established the relationship between them and the risk of childhood respiratory allergies with a dose-response pattern. As far as we know, to date, this is the only population-based study establishing the associations. To test the association, the analysis was further applied in subgroup whose allergen test was positive, and the similar results further enforced the evidence that maternal daily lifestyle choices are involved in childhood susceptibility to respiratory allergic diseases.

Although this is the first epidemiological study to explore the relationship between maternal lifestyle choice and offspring allergies, several potential explanations were in biological mechanisms to support the relationship. Several studies have demonstrated that early-life adverse exposure in utero can block the production of the immune response of fetal cytokines to Th1 type and affect the programming of fetal immune function [33, 38, 39]. Besides, increased stress and inflammatory response were put forward to explain the pathway. Sleep deprivation may lead to oxidative stress and inflammation, thus affecting early embryonic development [40]; while regular exercise can effectively buffer stress and prevent inflammation-related diseases through the mother-fetus connection [41]. Moreover, prenatal unhealthy behavioral exposure may also affect the microbiota of pregnant women, thereby transferring maternal bacteria to the fetus [42]. The imbalance of intestinal flora in infancy may lead to the deviation of immune function and allergic reactions [43].

It was impressed that respiratory allergies were mainly prevalent in males when their mothers were exposed to adverse lifestyle behaviors during pregnancy. After adjusting for socioeconomic factors, it turned out that the significance of maternal lifestyle in relation to childhood allergic diseases disappeared in females. This demonstrated that socio-economic factors did have an impact on the health effects of mothers on their offspring and that there were sex differences in these health effects. In a rat model, maternal high-fat diet during pregnancy was associated with neonatal cardiac dysfunction, reduced respiratory capacity and oxidative stress only in male offspring, indicating that adverse prenatal exposures impaired dynamism with sex-divergent characteristic [38]. A previous longitudinal study in the US also examined the sex-specific relationship between prenatal risk factors and offspring’s asthma outcomes, and the significant result was observed also only in males [44]. It has been recognized that male and female possessed different immune responses, especially in T-helper cells [39]. In referring to genetics, this pattern of inheritance may be linked to an X-linked gene, which is more likely to be exposed in boys with only one X-chromosome [45]. For example, IL-9 has impact on the development of the allergic disease by taking function in enhancing the synthesis of IgE and maturation of mast cell [46]. The IL-9 receptor gene, situated within the pseudoautosomal region of X and Y chromosomes, escaped X inactivation and its Y allele was also expressed [46, 47]. Another pro-inflammatory cytokine, IL-1β, is also been suspected of involvement in the pathogenesis of sex-specific allergic diseases in adult male asthma patients [48]. And the IL-1 family of molecules is among candidate genes of allergic susceptibility [49]. Sex hormones may also play a part, which respiratory genes could involve in the production of sex hormones [15]. Estrogen, the primary female sex hormone, has been regarded as an immune-stimulating factor [15]. While testosterone, as male sex hormones, tends to have immunosuppressive effects [15]. Nevertheless, the association was observed primarily in preschool children, suggesting that hormonal factors may not well explain the observed sex differences. Currently, the exact mechanism underlying has not been elucidated yet. Our results indicate the need for future studies to explore the association between prenatal factors and childhood asthma with consideration of possible sex-specific pathway.

This study has several limitations. First, information on maternal behavioral exposure was obtained from questionnaires, and thus the recall bias was inevitable. However, due to pregnancy was a special period, and China’s one-child policy in the past decades, the recall bias can be minimal. Second, a cross-sectional study was poor in determining causal links. However, the dependent variables and the outcome variables we analyzed were mothers’ lifestyles during pregnancy and their children’s respiratory allergies respectively, which had a time sequence, thus revealing a certain causal relationship. Third, allergic diseases were not diagnosed by professionals. However, the international standard ISAAC questionnaire was applied worldwide with quite good reliability and validity [22]. In addition, children with positive allergen tests were analyzed as a subgroup, which further verified our findings. Finally, even though many confounding factors were taken into account, residual confounding may remain. And environmental variables were obtained by questions, the field sampling data was unavailable. However, given that the results were broadly consistent across all the respiratory allergies in this study, the evidence for the link between maternal behavioral exposure and childhood allergic disease appeared to be robust.

## Conclusion

This study is the first to emphasize the association between maternal unhealthy behaviors such as lack of sleep, physical inactivity, and prolonged use of electronic devices during pregnancy and the risk of childhood allergies in a sex-specific manner. In particular, there appeared to have a clear dose-response relationship, the more unhealthy maternal behaviors, the greater risk of childhood respiratory allergies. However, this risk mainly affects males. Our findings should be of interventional significance in early-life since maternal lifestyle behaviors are modifiable.

## Supplementary information

**Additional file 1: Table S1.** Association of maternal behavioral factors during pregnancy with childhood respiratory allergies. **Table S2.** Association of maternal behavioral factors during pregnancy with childhood respiratory allergies stratified by gender and family allergic history. **Table S3.** Combined association of maternal behavioral factors during pregnancy with childhood respiratory allergies.

## Data Availability

The datasets used and/or analysed during the current study are available from the corresponding author on reasonable request.

## Abbreviations

SCAS: Shanghai Children Allergy Study
MLQ: Maternal Life-style Questionnaire
ISAAC: International Study of Asthma and Allergies in Childhood
KMO: Kaiser-Meyer-Olkin
SP: Screened Positive
SPT: Skin prick test
IgE: Immunoglobulin E
STP: Screened and Test Positive
OR: Odds Ratios
CI: Confidence Intervals
SPSS: Statistical Package for the Social Sciences
