# Supplementary material for: Effect of maternal sleep, physical activity and screen time during pregnancy on the risk of childhood respiratory allergies: a sex-specific study
Source: Respir Res. 2020 Sep 3;21:230. doi: 10.1186/s12931-020-01497-8 (PMC7650521; doi:10.1186/s12931-020-01497-8)
Supplement: Supplementary file 1 — Additional file 1: Table S1. Association of maternal behavioral factors during pregnancy with childhood respiratory allergies. Table S2. Association of maternal behavioral factors during pregnancy with childhood respiratory allergies stratified by gender and family allergic history. Table S3. Combined association of maternal behavioral factors during pregnancy with childhood respiratory allergies. [file 12931_2020_1497_MOESM1_ESM.docx]

| **Table S1** Association of maternal behavioral factors during pregnancy with childhood respiratory allergies | | | | | | | | | |
| --- | --- | --- | --- | --- | --- | --- | --- | --- | --- |
|  | **Asthma** | | | **Wheeze** | | | **Allergic Rhinitis** | | |
|  | OR (95% CI) | | | OR (95% CI) | | | OR (95% CI) | | |
|  | Crude model | Adjusted Model I | Adjusted Model II | Crude model | Adjusted Model I | Adjusted Model II | Crude model | Adjusted Model I | Adjusted Model II |
| **Male** | | | | | | | | | |
| **Sleep duration** |  |  |  |  |  |  |  |  |  |
| <8 vs. ≥8 hrs./day | **1.401 (1.140-1.721)^***^** | **1.296 (1.049-1.601) ^*^** | **1.268 (1.024-1.571)^*^** | **1.354 (1.109-1.654) ^**^** | **1.251 (1.018-1.537)^*^** | 1.224 (0.993-1.508) | **1.441 (1.205-1.722)^***^** | **1.289 (1.065-1.560)^**^** | **1.282 (1.057-1.554)^*^** |
| **Physical activity** |  |  |  |  |  |  |  |  |  |
| <1 vs. ≥1 hrs./day | **1.423 (1.105-1.831)^**^** | 1.265 (0.975-1.642) | 1.237 (0.950-1.610) | **1.491 (1.166-1.906) ^***^** | **1.328 (1.030-1.712) ^*^** | **1.294 (1.000-1.673) ^*^** | **1.651 (1.326-2.056)^***^** | **1.460 (1.155-1.845)^**^** | **1.458 (1.152-1.847)^**^** |
| **Screen exposure** |  |  |  |  |  |  |  |  |  |
| ≥2 vs. <2hrs./day | **1.523 (1.203-1.926)^***^** | 1.248 (0.974-1.598) | **1.298 (1.011-1.667) ^*^** | **1.621 (1.289-2.039) ^***^** | **1.327 (1.042-1.690) ^*^** | **1.382 (1.082-1.764) ^*^** | **1.358 (1.119-1.649)^**^** | 1.063 (0.859-1.316) | 1.083 (0.873-1.343) |
| **Female** | | | | | | | | | |
| **Sleep duration** |  |  |  |  |  |  |  |  |  |
| <8 vs. ≥8 hrs./day | **1.334 (1.046-1.701)^*^** | 1.234 (0.962-1.582) | 1.215 (0.946-1.561) | **1.271 (1.007-1.604)^*^** | 1.169 (0.920-1.485) | 1.140 (0.895-1.453) | 1.166 (0.935-1.455) | 1.001 (0.792-1.264) | 0.966 (0.763-1.224) |
| **Physical activity** |  |  |  |  |  |  |  |  |  |
| <1 vs. ≥1 hrs./day | 1.018 (0.768-1.349) | 0.926 (0.693-1.237) | 0.925 (0.691-1.239) | 1.051 (0.802-1.376) | 0.949 (0.719-1.254) | 0.938 (0.708-1.244) | 1.173 (0.906-1.519) | 1.018 (0.775-1.337) | 0.990 (0.751-1.304) |
| **Screen exposure** |  |  |  |  |  |  |  |  |  |
| ≥2 vs. <2hrs./day | **1.429 (1.075-1.901)^**^** | 1.282 (0.949-1.733) | 1.287 (0.950-1.7433) | **1.471 (1.120-1.931) ^**^** | 1.281 (0.960-1.708) | 1.295 (0.968-1.732) | **1.362 (1.062-1.747)^*^** | 1.027 (0.785-1.344) | 1.063 (0.810-1.395) |

OR, odds ratios; CI, confidence interval

* *p* < 0.05, ***p* < 0.01, ****p* < 0.001.

Model I adjusted for child's age, childhood overweight/obesity, child’s sleep duration on weekdays, child’s sleep duration on weekends, child’s exercise frequency, children’s screen exposure frequency, family income, family structure, mother’s education, father’s education, family history of allergic diseases.

Model II further adjusted for gestational weeks, delivery mode, full breastfeeding, maternal smoking exposure, maternal drinking, gestational hypertension, gestational diabetes, gestational anemia, pre-pregnancy overweight/obesity, mother's age at delivery, farmlands and orchards near house, chemical discharging near house, factories emitting smoke near house, large garbage dump near house.

| **Table S2** Association of maternal behavioral factors during pregnancy with childhood respiratory allergies stratified by gender and family allergic history | | | | | | | | |  |
| --- | --- | --- | --- | --- | --- | --- | --- | --- | --- |
|  | **Asthma** | | | **Wheeze** | | | **Allergic Rhinitis** | | |
|  | OR (95% CI) | | | OR (95% CI) | | | OR (95% CI) | | |
|  | Crude Model | Adjusted Model I | Adjusted Model II | Crude Model | Adjusted Model I | Adjusted Model II | Crude Model | Adjusted Model I | Adjusted Model II |
| **Male** |  |  |  |  |  |  |  |  |  |
| **First-degree relative with allergies** |  |  |  |  |  |  |  |  |  |
| **Sleep duration** |  |  |  |  |  |  |  |  |  |
| <8 vs. ≥8 hrs./day | 1.213 (0.879-1.675) | 1.207 (0.871-1.673) | 1.138 (0.812-1.595) | 1.190 (0.869-1.629) | 1.186 (0.863- 1.629) | 1.127 (0.812-1.565) | 1.025 (0.766-1.372) | 1.011 (0.750-1.363) | 1.001 (0.738-1.358) |
| **Physical activity** |  |  |  |  |  |  |  |  |  |
| <1 vs. ≥1 hrs./day | **1.660 (1.067-2.583)^*^** | **1.695 (1.078-2.665)^*^** | **1.676 (1.054-2.665^)*^** | **1.769 (1.150-2.721)^**^** | **1.802 (1.160-2.799)^**^** | **1.777 (1.132-2.789)^**^** | 1.408 (0.980-2.024) | 1.368 (0.938-1.996) | 1.388 (0.945-2.038) |
| **Screen exposure** |  |  |  |  |  |  |  |  |  |
| ≥2 vs. <2hrs./day | 1.085 (0.734-1.604) | 1.018 (0.679-1.527) | 1.156 (0.762-1.755) | 1.189 (0.810-1.745) | 1.144 (0.769-1.702) | 1.301 (0.864-1.958) | 0.888 (0.631-1.250) | 0.889 (0.618-1.280) | 0.873 (0.603-1.265) |
| **First-degree relative without allergies** |  |  |  |  |  |  |  |  |  |
| **Sleep duration** |  |  |  |  |  |  |  |  |  |
| <8 vs. ≥8 hrs./day | **1.426 (1.084-1.876)^*^** | **1.363 (1.031-1.800)^*^** | **1.349 (1.017-1.789)^*^** | **1.358 (1.039-1.775) ^*^** | 1.295 (0.986-1.700) | 1.276 (0.968-1.681) | **1.650 (1.297-2.099)^***^** | **1.526 (1.194-1.950)^***^** | **1.499 (1.169-1.923)^**^** |
| **Physical activity** |  |  |  |  |  |  |  |  |  |
| <1 vs. ≥1 hrs./day | 1.192 (0.872-1.629) | 1.106 (0.803-1.523) | 1.106 (0.800-1.528) | 1.236 (0.912-1.676) | 1.143 (0.836-1.563) | 1.142 (0.832-1.566) | **1.637 (1.216-2.203)^***^** | **1.488 (1.097-2.018)^*^** | **1.465 (1.078-1.993)^*^** |
| **Screen exposure** |  |  |  |  |  |  |  |  |  |
| ≥2 vs. <2hrs./day | **1.582 (1.167-2.144)^**^** | 1.357 (0.988-1.864) | 1.365 (0.991-1.879) | **1.651 (1.227-2.220)^***^** | **1.397 (1.025-1.901)^*^** | **1.416 (1.036-1.934)^*^** | **1.361 (1.051-1.763)^*^** | 1.140 (0.868-1.496) | \| 1.176 (0.893-1.549) \| \| --- \| |
| **Female** |  |  |  |  |  |  |  |  |  |
| **First-degree relative with allergies** |  |  |  |  |  |  |  |  |  |
| **Sleep duration** |  |  |  |  |  |  |  |  |  |
| <8 vs. ≥8 hrs./day | 1.227 (0.835-1.803) | 1.180 (0.799-1.744) | 1.152 (0.773-1.717) | 1.059 (0.732-1.532) | 1.016 (0.697-1.480) | 0.968 (0.659-1.423) | 1.063 (0.763-1.481) | 0.991 (0.704-1.396) | 0.952 (0.669-1.356) |
| **Physical activity** |  |  |  |  |  |  |  |  |  |
| <1 vs. ≥1 hrs./day | 0.972 (0.601-1.572) | 0.855 (0.518-1.412) | 0.847 (0.505-1.422) | 0.987 (0.626-1.556) | 0.903 (0.561-1.455) | 0.911 (0.557-1.490) | 1.451 (0.942-2.234) | 1.229 (0.779-1.939) | 1.214 (0.760-1.938) |
| **Screen exposure** |  |  |  |  |  |  |  |  |  |
| ≥2 vs. <2hrs./day | 1.456 (0.814-2.603) | 1.373 (0.753-2.502) | 1.541 (0.832-2.854) | 1.546 (0.890-2.687) | 1.450 (0.818-2.573) | 1.625 (0.899-2.937) | 1.242 (0.782-1.974) | 1.167 (0.716-1.903) | 1.135 (0.687-1.875) |
| **First-degree relative without allergies** |  |  |  |  |  |  |  |  |  |
| **Sleep duration** |  |  |  |  |  |  |  |  |  |
| <8 vs. ≥8 hrs./day | 1.299 (0.945-1.786) | 1.271 (0.919-1.757) | 1.216 (0.875-1.689) | 1.310 (0.965-1.779) | 1.281 (0.938-1.751) | 1.222 (0.890-1.678) | 1.057 (0.768-1.454) | 1.004 (0.726-1.387) | 0.958 (0.690-1.330) |
| **Physical activity** |  |  |  |  |  |  |  |  |  |
| <1 vs. ≥1 hrs./day | 0.984 (0.692-1.400) | 0.917 (0.640-1.313) | 0.938 (0.652-1.349) | 1.019 (0.724-1.434) | 0.934 (0.659-1.323) | 0.937 (0.657-1.335) | 0.921 (0.659-1.287) | 0.883 (0.628-1.242) | 0.858 (0.607-1.211) |
| **Screen exposure** |  |  |  |  |  |  |  |  |  |
| ≥2 vs. <2hrs./day | 1.210 (0.866-1.691) | 1.217 (0.856-1.729) | 1.197 (0.839-1.706) | 1.204 (0.874-1.659) | 1.189 (0.849-1.666) | 1.176 (0.836-1.655) | 1.018 (0.746-1.389) | 0.945 (0.683-1.307) | 0.991 (0.713-1.377) |

OR, odds ratios; CI, confidence interval; Ref, reference; ^*^ *p* < 0.05, ^**^*p*<0.01, ^***^*p*<0.001.

Model I adjusted for child's age, childhood overweight/obesity, child’s sleep duration on weekdays, child’s sleep duration on weekends, child’s exercise frequency, children’s screen exposure frequency, family income, family structure, mother’s education, father’s education, family history of allergic diseases.

Model II further adjusted for gestational weeks, delivery mode, full breastfeeding, maternal smoking exposure, maternal drinking, maternal pre-pregnancy BMI, gestational hypertension, gestational diabetes, gestational anemia, pre-pregnancy overweight/obesity, mother's age at delivery, farmlands and orchards near house, chemical discharging near house, factories emitting smoke near house, large garbage dump near house.

| **Table S3** Combined association of maternal behavioral factors during pregnancy with childhood respiratory allergies | | | | | | | | | | | | | |
| --- | --- | --- | --- | --- | --- | --- | --- | --- | --- | --- | --- | --- | --- |
|  |  | **Asthma** | | |  | **Wheeze** | | |  | **Allergic rhinitis** | | | |
|  | N (%) | OR (95% CI) | | | N (%) | OR (95% CI) | | | N (%) | OR (95% CI) | | | |
|  |  | Crude model | Adjusted Model I | Adjusted Model II |  | Crude model | Adjusted Model I | Adjusted Model II |  | Crude model | Adjusted Model I | | Adjusted Model II^b^ |
| **Male** | | | | | | | | | | | | | |
| **0** | 10  (1.9%) | Ref | Ref | Ref | 11  (1.9%) | Ref | Ref | Ref | 22  (2.8%) | Ref | | Ref | Ref |
| **1 vs. 0** | 124  (23.4%) | **2.550**  **(1.265-5.139)^**^** | **2.378**  **(1.169-4.839)^*^** | **2.301**  **(1.127-4.699)^*^** | 134  (22.9%) | **2.747**  **(1.365-5.528)^**^** | **2.551**  **(1.255-5.187)^*^** | **2.489**  **(1.221-5.078)^*^** | 168  (21.0%) | 1.517  (0.919-2.504) | | 1.452  (0.857-2.460) | 1.468  (0.866-2.489) |
| **2 vs. 0** | 276  (52.0%) | **2.991**  **(1.505-5.943)^**^** | **2.378**  **(1.184-4.778)^*^** | **2.319**  **(1.150-4.677)^*^** | 308  (52.7%) | **3.412**  **(1.719-6.773)^***^** | **2.700**  **(1.345-5.421)^**^** | **2.636**  **(1.309-5.308)^**^** | 440  (55%) | **2.269**  **(1.399-3.681)^***^** | | **1.804**  **(1.083-3.007)^*^** | **1.837**  **(1.101-3.065)^*^** |
| **3 vs. 0** | 121  (22.8%) | **4.606**  **(2.277-9.318)^***^** | **3.388**  **(1.653-6.943)^***^** | **3.281**  **(1.595-6.750)^**^** | 131  (22.4%) | **5.069**  **(2.509-10.239)^***^** | **3.708**  **(1.812-7.590)^***^** | **3.612**  **(1.759-7.417)^***^** | 170  (21.3%) | **3.018**  **(1.819-5.007)^***^** | | **2.129**  **(1.245-3.638)^**^** | **2.153**  **(1.258-3.684)^**^** |
| **Female** | | | | | | | | | | | | | |
| **0** | 13  (3.4%) | Ref | Ref | Ref | 13  (3.1%) | Ref | Ref | Ref | 13  (2.6%) | Ref | | Ref | Ref |
| **1 vs. 0** | 76  (20.2%) | 0.848  (0.453-1.589) | 0.781  ().413-1.476) | 0.762  (0.401-1.448) | 86  (20.3%) | 0.983  (0.527-1.831) | 0.893  (0.474-1.683) | 0.857  (0.453-1.622) | 113  (15.1%) | 1.514  (0.804-2.849) | | 1.279  (0.667-2.453) | 1.222  (0.633-2.356) |
| **2 vs. 0** | 211  (56.0%) | 1.194  (0.658-2.167) | 0.967  (0.523-1.787) | 0.943  (0.508-1.750) | 242  (57.1%) | 1.405  (0.776-2.545) | 1.102  (0.597-2.035) | 1.050  (0.566-1.947) | 282  (55.7%) | 1.796  (0.973-3.314) | | 1.190  (0.629-2.250) | 1.129  (0.594-2.146) |
| **3 vs. 0** | 77  (20.4%) | 1.416  (0.755-2.655) | 1.106  (0.578-2.118) | 1.073  (0.558-2.064) | 83  (19.6%) | 1.554  (0.831-2.906) | 1.172  (0.613-2.240) | 1.112  (0.579-2.135) | 98  (19.4%) | **2.099**  **(1.107-3.977)*** | | 1.252  (0.642-2.440) | 1.177  (0.600-2.308) |

OR, odds ratios; CI, confidence interval; Ref, reference.

^*^ *p* < 0.05, ^**^*p* < 0.01, ^***^*p* < 0.001.

Maternal risk behavioral factors included sleep duration less than 8 hours, physical activity less than 1 hour/day, screen exposure more than 2 hours/day; “0” demonstrated that none of three risk factors were occurred. “1” demonstrated that one of these three risk factors was occurred; “2 ” demonstrated that two of these three risk factors were occurred; and “3” demonstrated that these three risk factors were all occurred.

Model I adjusted for child's age, childhood overweight/obesity, child’s sleep duration on weekdays, child’s sleep duration on weekends, child’s exercise frequency, children’s screen exposure frequency, family income, family structure, mother’s education, father’s education, family history of allergic diseases.

Model II further adjusted for gestational weeks, delivery mode, full breastfeeding, maternal smoking exposure, maternal drinking, maternal pre-pregnancy BMI, gestational hypertension, gestational diabetes, gestational anemia, pre-pregnancy overweight/obesity, mother's age at delivery, farmlands and orchards near house, chemical discharging near house, factories emitting smoke near house, large garbage dump near house.
